# Fish, Fish-Derived n-3 Fatty Acids, and Risk of Incident Atrial Fibrillation in the Atherosclerosis Risk in Communities (ARIC) Study

**DOI:** 10.1371/journal.pone.0036686

**Published:** 2012-05-03

**Authors:** Noelle N. Gronroos, Alanna M. Chamberlain, Aaron R. Folsom, Elsayed Z. Soliman, Sunil K. Agarwal, Jennifer A. Nettleton, Alvaro Alonso

**Affiliations:** 1 Division of Epidemiology and Community Health, School of Public Health, University of Minnesota, Minneapolis, Minnesota, United States of America; 2 Department of Health Sciences Research, Mayo Clinic, Rochester, Minnesota, United States of America; 3 Epidemiological Cardiology Research Center (EPICARE), Wake Forest University School of Medicine, Winston Salem, North Carolina, United States of America; 4 University of North Carolina at Chapel Hill, Chapel Hill, North Carolina, United States of America; 5 Division of Epidemiology, Human Genetics and Environmental Sciences, School of Public Health, University of Texas Health Science Center at Houston, Houston, Texas, United States of America; University Medical Center Utrecht, The Netherlands

## Abstract

**Background:**

Results of observational and experimental studies investigating the association between intake of long-chain n-3 polyunsaturated fatty acids (PUFAs) and risk of atrial fibrillation (AF) have been inconsistent.

**Methods:**

We studied the association of fish and the fish-derived n-3 PUFAs eicosapentaenoic acid (EPA) and docosahexaenoic acid (DHA) with the risk of incident AF in individuals aged 45–64 from the Atherosclerosis Risk in Communities (ARIC) cohort (n = 14,222, 27% African Americans). Intake of fish and of DHA and EPA were measured via food frequency questionnaire. Plasma levels of DHA and EPA were measured in phospholipids in a subset of participants (n = 3,757). Incident AF was identified through the end of 2008 using ECGs, hospital discharge codes and death certificates. Cox proportional hazards regression was used to estimate hazard ratios of AF by quartiles of n-3 PUFAs or by fish intake.

**Results:**

During the average follow-up of 17.6 years, 1,604 AF events were identified. In multivariable analyses, total fish intake and dietary DHA and EPA were not associated with AF risk. Higher intake of oily fish and canned tuna was associated with a nonsignificant lower risk of AF (p for trend = 0.09). Phospholipid levels of DHA+EPA were not related to incident AF. However, DHA and EPA showed differential associations with AF risk when analyzed separately, with lower risk of AF in those with higher levels of DHA but no association between EPA levels and AF risk.

**Conclusions:**

In this racially diverse sample, dietary intake of fish and fish-derived n-3 fatty acids, as well as plasma biomarkers of fish intake, were not associated with AF risk.

## Introduction

Consumption of fish and fish-derived n-3 PUFAs has been shown to reduce the risk of cardiovascular disease [1], [2], particularly sudden cardiac death [3]. Similarly, consumption of fish and n-3 PUFAs have been associated with more favorable heart rate variability indices [4], and reduced risk of ventricular arrhythmias [5] and all-cause mortality post myocardial infarction (MI) [6].

Intake of fish and the fish-derived n-3 fatty acids DHA and EPA may also reduce the risk of atrial fibrillation (AF), a common cardiac arrhythmia associated with increased stroke and cardiovascular morbidity and mortality, which affects more than 2 million Americans [7]. However, studies investigating the association between fish-derived n-3 PUFAs and incident AF have had inconsistent results [8], [9], [10], [11], [12], [13], [14], [15], [16].

These observed inconsistencies may be due, in part, to differences in dietary assessment methods, absolute quantities of fish consumed, and dietary patterns associated with fish consumption across different study populations. Specifically, measurement error of fish intake, or any other dietary exposure, poses a concerning issue since self-reported information is subject to recall bias and biomarkers of fish intake (such as plasma levels of DHA and EPA) only reflect exposure over a limited period of time and are affected by multitude of metabolic processes [17]. Using a combination of self-reported and objective measures (biomarkers) of fish consumption in the same population might provide a more accurate representation of the association with AF risk taking advantage of the strengths of each method. Therefore, we used both self-reported measures of fish and dietary EPA and DHA intake (derived from food frequency questionnaires (FFQ)) and phospholipid measures of EPA and DHA to test the hypothesis that fish and EPA and DHA are inversely associated with the risk of incident AF in the Atherosclerosis Risk in Communities (ARIC) Study, a population based cohort of middle aged American men and women.

## Methods

### Study Population

The ARIC study has been described previously [18]. Briefly, ARIC is a prospective study of cardiovascular disease including 15,792 men and women aged 45–64 years of age at baseline from four US communities: Forsyth County, NC; Jackson, MS; Minneapolis suburbs, MN; and Washington County, MD. Baseline data were collected in 1987–89. Three additional exams were done at 3-year intervals (1990–92, 1993–95, 1996–98). Follow-up for cardiovascular outcomes is available through 2008. The ARIC study has been approved by the Institutional Review Boards (IRB) of all participating institutions, including the IRBs of the University of Minnesota, Johns Hopkins University, University of North Carolina, University of Mississippi Medical Center, and Wake Forest University. All participants gave written informed consent in each one of the study visits.

### AF ascertainment

Incident cases of atrial fibrillation through December 31, 2008 were identified from three sources: hospital discharge codes (*International Classification of Diseases*, *Ninth Revision* (ICD-9) codes 427.31 and 427.32), electrocardiograms (ECGs) performed during follow-up exams, and death certificates (ICD-9 code 437.3 or ICD-10 code I48) [19]. The positive predictive value of hospital discharge codes for the diagnosis of incident AF, as determined after review of hospital discharge summaries in a sample of ARIC participants, was 89% [20].

### Exposure assessment

Dietary intake of fish and the fish-derived PUFAs DHA and EPA was ascertained using both self-report and biomarkers. Participants reported their usual intake of different types of fish via a food frequency questionnaire (FFQ). Additionally, blood plasma levels of DHA and EPA were measured in a subset of participants.

**Fish intake via FFQ:** Participants' usual dietary intake was assessed by an interviewer-administered, 66-item FFQ. The FFQ was a slightly modified version of the instrument developed by Willett et al [21]. The FFQ was administered to all subjects at baseline (1987–1989) and Exam 3 (1993–1995). For each food, participants were asked to report the frequency of consumption over the past year in 9 categories, ranging from “never or less than once per month” to “>6 times per day.” Interviewers used food models to help with portion size estimation. Fish and other seafood intake was assessed through 4 questionnaire items: (1) 3–4 ounces of canned tuna fish; (2) 3–5 ounces of dark meat fish such as salmon, mackerel, swordfish, sardines, and bluefish; (3) 3–5 ounces of other fish such as cod, perch, catfish, etc.; and (4) shrimp, lobster, scallops as a main dish. FFQ responses were translated into servings per week and subsequently categorized into four categories: none, less than 1, 1–2, and more than two. The FFQ did not collect information on fish preparation method.

**DHA and EPA via FFQ:** Nutrient values for each food were obtained from the Harvard database [21] and daily intake of nutrients was calculated by multiplying the nutrient content of each food in the portion specified by the frequency of daily consumption and then summing the results. This calculation yielded consumption of EPA and DHA in grams/day. Fish-derived n-3 fatty acid intake in visits 1 and 3 were significantly correlated (r = 0.47, p<0.0001, adjusted for age, race, and sex)

**DHA and EPA in Plasma:** Fatty acids levels were measured in plasma samples from the Minnesota field center participants (n = 3,757) at baseline. Fatty acids were measured in plasma cholesterol esters and phospholipids using gas chromatography, yielding measures of plasma DHA and EPA as a percentage of total fatty acids. The fatty acid profile of cholesterol esters reflects medium-term (weeks) dietary intake of fatty acids while phospholipids reflect intake over a slightly longer duration (weeks to months) [22]. Only phospholipid measurements were used for the present analysis.

Previous analyses in ARIC have shown that plasma DHA and EPA measures correlate with dietary intake (as measured via a food frequency questionnaire) with correlation coefficients ranging from 0.20 (EPA) to 0.42 (DHA) [23].

**Assessment of other variables:** Cigarette smoking status and amount, exercise amount, systolic blood pressure, LDL and HDL cholesterols, alcohol intake, antihypertensive medication use, diabetes status, anti-hyperglycemic medication use, weight, height, and educational status were measured at baseline using standardized methods [18]. Diabetes was defined as fasting blood glucose ≥126 mg/dl, use of anti-hyperglycemic medications, or self-reported history of physician-diagnosed diabetes. ECG-diagnosed left ventricular hypertrophy (LVH) was considered present if the Cornell voltage was >28 mm in men or >22 mm in women [24]. History of coronary heart disease (CHD) at baseline was defined as one of the following: a self-reported history of a physician-diagnosed myocardial infarction; evidence of previous myocardial infarction in the baseline ECG; history of previous heart or arterial surgery, including angioplasty or coronary bypass.

Participants with prevalent AF at baseline, who reported a race other than white or black, and those with implausible calorie intakes (<700 or >4500 for men, <500 or >3500 for women) were excluded. Participants with missing values for diabetes, prevalent CHD, or LVH at baseline were imputed as having “no disease.” Participants missing exposure values or other covariates were excluded from analysis.

### Statistical analysis

Cox proportional hazards regression models were used to estimate hazard ratios (HRs) of incident AF by level of fish consumption (0, <1, 1–2, 2+ servings/week), quartiles of DHA+EPA intake, and quartiles of phospholipid DHA+EPA. Exposure status was a time-dependent covariate with baseline (visit 1) dietary data used as the exposure for the period between baseline and visit 3, and the average intake of visits 1 and 3 afterwards. If visit 3 data were not available then visit 1 data were used over the entire follow-up. The multivariable-adjusted models included the following potential confounders as measured at baseline: center, age, sex, race, BMI, total calories, alcohol intake (grams/day), saturated fat intake (grams/day), fiber intake (grams/day), vitamin C intake (mg/day), education level (less than high school, high school, more than high school), cigarette smoking status (current, former, never) and amount (pack years), LDL cholesterol, HDL cholesterol, systolic blood pressure, use of hypertensive medications, diabetes, LVH, and prevalent CHD. Tests of linear trend were conducted by assigning the median values for each category of exposure variable and modeling as a continuous variable. To assess potential U-shaped associations, tests of quadratic trend were done by using the linear trend values and adding a quadratic term. To determine whether method of AF ascertainment affected our results, we repeated all analyses including only AF events identified in study ECGs. We used the residual method to adjust for total energy intake in analyses considering dietary EPA and DHA as the main exposure [25].

Additional analyses were conducted combining the dietary data with the biomarker data. Multivariable Cox proportional hazard models were run using three different exposures: (1) subjects in the highest tertiles of both FFQ-derived residual-adjusted intake and phospholipid measures of DHA+EPA vs. those in lowest tertiles vs. all others (3-level categorical variable); (2) Howe's method [26] with three categories (tertiles); and (3) Howe's method with n categories (n = 3,743). Howe's method ranks individuals by categories of self-reported diet intake (DHA+EPA), then again by biomarkers (DHA+EPA), then sums the two ranks [26]. Thus, Howe's method for three categories has a range of a combined rank from 2 (lowest tertile for both dietary and phospholipid exposures) to 6 (highest tertile for both dietary and phospholipid exposures). Howe's method for n categories ranks all subjects in sequential order from lowest intake of DHA+EPA to highest.

Tests of the proportional hazards assumption were evaluated using a time*exposure interaction term. Sex by exposure and race by exposure interactions were also tested using a sex*exposure and race*exposure interaction term. All p-values were 2-tailed. Data were analyzed with SAS 9.2 for Windows (SAS Corp, Cary, NC).

## Results

Baseline characteristics of the 14,222 eligible participants by categories of fish intake (all ARIC field centers), and the 3,757 eligible participants by quartiles of DHA+EPA phospholipid levels (Minnesota field center only) are shown in Table 1. Those who consumed more fish tended to be older, female, and have a more adverse cardiovascular risk profile. During the average follow-up time of 17.6 years (249,775 person-years), 1,604 AF events were identified. The proportional hazards assumption was not violated.

**Table 1:** Baseline characteristics of ARIC participants (n = 14,222), 1987–1989.

	Serving of fish/week			
	0	<1	1–2	>2
Number of Subjects (%)	612 (4.3)	2641 (18.6)	5686 (40)	5283 (37.1)
Age	55 (6.0)	54.2 (5.8)	54.2 (5.7)	54 (5.7)
Sex (men)	48.0	51.7	54.5	58.9
Race (White)	88.9	89.6	76.8	64.5
BMI	27.2 (5.4)	27.1 (4.8)	27.5 (5.3)	28.2 (5.5)
Hypertension	28.6	28.9	34.2	37.2
Current smoking	29.7	25.2	26.7	24.5
Diabetes	10.8	9.4	11.2	12.9
ECG defined LVH	1.5	1.8	2	2.3
CHD	5.4	3.9	4.6	5.2
Alcohol intake (g/day)	5.6 (14.1)	6.2 (13.8)	6.4 (13.6)	5.8 (13.3)
Total energy intake (Kcal/day)	1504.4 (605.9)	1478.9 (572.4)	1561.8 (564.2)	1747.3 (611.0)
Fish-derived n-3 fatty acids (g/day)	0.02 (0.02)	0.08 (0.05)	0.19 (0.09)	0.48 (0.34)
Fiber (g/day)	15.6 (8.7)	14.9 (7.2)	16.4 (7.3)	19.7 (9)
Vitamin C (mg/day)	104.5 (79.9)	99.6 (73.9)	116.4 (77.7)	142.8 (91.6)
Saturated Fat (g/day)	21.8 (11)	21.3 (10.5)	21.9 (10.5)	22.4 (10.8)

	Quartile of DHA+EPA (Minnesota participants only, n = 3,817)			
	Q1	Q2	Q3	Q4
Range (% total fatty acid)	0.73–2.7	2.71–3.2	3.21–3.82	3.83–12.19
Number of Subjects (%)	410 (21)	483 (24.7)	550 (28.1)	511 (26.2)
Age	53.5 (5.6)	53.9 (5.7)	53.9 (5.6)	54.4 (5.6)
Sex (men)	55.68	48.51	41.8	46.15
BMI	26.6 (4.4)	27.5 (4.9)	27.3 (4.7)	26.7 (4.3)
Hypertension	24.86	24.31	27.09	25.29
Current smoking	34.38	21.86	18.84	14.12
Diabetes	6.16	8	7.3	7.38
ECG defined LVH	1.19	0.96	0.74	0.95
CHD	3.89	4.05	4.76	4.95
Alcohol intake (g/day)	9.8 (15.7)	8.7 (14.7)	7.6 (14.7)	7.4 (12)
Fiber (g/day)	15.5 (7.4)	15.8 (7.3)	16.1 (7.1)	17.4 (7.4)
Vitamin C (mg/day)	103.7 (72.2)	109.5 (71.4)	109.6 (62)	119.4 (68.9)
Saturated Fat (g/day)	24.9 (11.2)	23.8 (11.3)	22.1 (9.7)	20.7 (9.8)

Values are % for categorical variables and mean (SD) for continuous variables. BMI: Body mass index. CHD: Coronary heart disease. DHA: Docosahexaenoic acid. ECG: Electrocardiogram. EPA: Eicosapentanoic acid.

### Risk of AF by Fish Intake Categories

Overall, fish intake was not associated with the incidence of AF (table 2). The multivariable HR (95% CI) of AF in those consuming >2 servings of fish/week was 1.00 (0.81–1.24) compared to those not eating any fish (p for trend = 0.15). Further categorization of individuals with >2 serving of fish/week into >2–3, >3–4, >4–5, and >5 servings/week did not show evidence of an association (data not shown). Results were similar for canned tuna (HR 0.85, 95% CI: 0.68–1.06), oily fish (HR 0.83, 95% CI: 0.58–1.21), other fish (HR 0.98, 95% CI: 0.76–1.27), and shellfish (HR 0.81, 95% CI: 0.38–1.71), comparing >2 servings/week vs. no intake. An analysis combining canned tuna and oily fish showed a non-significant lower AF risk in those with >2 servings/week vs no intake (HR 0.86, 95% CI 0.72–1.03, p for trend = 0.09). Results did not materially change when we included only AF cases identified from study ECGs.

**Table 2:** Hazard ratios (95% confidence intervals) of atrial fibrillation by fish intake categories, ARIC, 1987–2008.

Total Fish (servings/week)	0	<1	1–2	>2	P for trend
AF cases	66	309	679	550	
Person-years	10,108	47,073	98,733	93,861	
Model 1 [HR (95% CI)]	1 (ref.)	1.04 (0.84, 1.29)	1.12 (0.92, 1.35)	0.99 (0.80, 1.21)	0.37
Model 2 [HR (95% CI)]	1 (ref.)	1.13 (0.91, 1.40)	1.16 (0.95, 1.40)	1.00 (0.81, 1.24)	0.15

Canned Tuna (servings/week)	0	<1	1–2	>2	
AF cases	394	792	326	92	
Person-years	53,825	125,261	52,237	18,453	
Model 1 [HR (95% CI)]	1 (ref.)	0.97 (0.86, 1.10)	1.01 (0.87, 1.17)	0.94 (0.76, 1.17)	0.77
Model 2 [HR (95% CI)]	1 (ref.)	0.98 (0.87, 1.11)	0.97 (0.84, 1.13)	0.85 (0.68, 1.06)	0.17

Oily Fish (servings/week)	0	<1	1–2	>2	
AF cases	664	768	148	24	
Person-years	108,509	114,245	22,226	4,795	
Model 1 [HR (95% CI)]	1 (ref.)	1.04 (0.93, 1.15)	0.99 (0.83, 1.18)	0.89 (0.61, 1.28)	0.67
Model 2 [HR (95% CI)]	1 (ref.)	1.03 (0.92, 1.14)	0.97 (0.81, 1.16)	0.83 (0.58, 1.21)	0.44

Canned Tuna + Oily Fish (servings/week)	0	<1	1–2	>2	
AF cases	425	429	572	178	
Person-years	60,541	66,681	89,598	32,955	
Model 1 [HR (95% CI)]	1 (ref.)	0.96 (0.84–1.09)	0.97 (0.85–1.10)	0.90 (0.76–1.07)	0.27
Model 2 [HR (95% CI)]	1 (ref.)	0.98 (0.85–1.12)	0.95 (0.83–1.08)	0.86 (0.72–1.03)	0.09

Other Fish (servings/week)	0	<1	1–2	>2	
AF cases	377	842	321	64	
Person-years	56,467	128,878	52,975	11,456	
Model 1 [HR (95% CI)]	1 (ref.)	1.03 (0.91, 1.17)	0.96 (0.82, 1.12)	0.97 (0.76, 1.26)	0.66
Model 2 [HR (95% CI)]	1 (ref.)	1.08 (0.96, 1.23)	0.98 (0.84, 1.14)	0.98 (0.76, 1.27)	0.68

Shellfish (servings/week)	0	<1	1–2	>2	
AF cases	846	673	77	8	
Person-years	129,037	107,438	11,716	1,584	
Model 1 [HR (95% CI)]	1 (ref.)	1.01 (0.91, 1.12)	1.19 (0.96, 1.49)	0.87 (0.41, 1.83)	0.51
Model 2 [HR (95% CI)]	1 (ref.)	1.04 (0.93, 1.15)	1.16 (0.92, 1.46)	0.81 (0.38, 1.71)	0.54

CI: Confidence interval. HR: Hazard ratio. Model 1: adjusted for age, sex, and race; Model 2: adjusted for center, age, race, sex, energy intake, body mass index, education, exercise levels, smoking status and amount, alcohol intake, LDL cholesterol, HDL cholesterol, use of cholesterol lowering medications, systolic blood pressure, use of antihypertensive medications, diabetes, coronary heart disease, and ECG-defined left ventricular hypertrophy.

### Risk of AF by DHA and EPA Intake

Intake of DHA+EPA was not associated with the incidence of AF, with a HR (95% CI) of 0.92 (0.79–1.07) comparing extreme quartiles (Table 3). Similar results were found when dietary DHA and EPA were analyzed separately.

**Table 3:** Hazard ratios (95% confidence interval) of atrial fibrillation by categories of DHA+EPA intake, ARIC, 1987–2008.

Dietary DHA+EPA (Quartiles)	Q1	Q2	Q3	Q4	P for trend
AF cases	402	427	409	366	
Person-years	61,943	62,339	62,270	63,223	
Model 1 [HR (95% CI)]	1 (ref.)	1.03 (0.90, 1.19)	1.06 (0.91, 1.23)	0.95 (0.82, 1.10)	0.42
Model 2 [HR (95% CI)]	1 (ref.)	1.04 (0.90, 1.20)	1.06 (0.91, 1.23)	0.92 (0.79, 1.07)	0.21

Dietary DHA (Quartiles)	Q1	Q2	Q3	Q4	
AF cases	404	428	410	362	
Person-years	61,750	62,584	62,134	63,307	
Model 1 [HR (95% CI)]	1 (ref.)	1.06 (0.92, 1.22)	1.05 (0.90, 1.22)	0.97 (0.83, 1.12)	0.48
Model 2 [HR (95% CI)]	1 (ref.)	1.06 (0.92, 1.23)	1.05 (0.90, 1.22)	0.93 (0.80, 1.09)	0.21

Dietary EPA (Quartiles)	Q1	Q2	Q3	Q4	
AF cases	412	418	392	382	
Person-years	61,962	62,298	62,701	62,815	
Model 1 [HR (95% CI)]	1 (ref.)	1.07 (0.93, 1.23)	1.00 (0.86, 1.16)	0.96 (0.83, 1.12)	0.41
Model 2 [HR (95% CI)]	1 (ref.)	1.05 (0.91, 1.22)	1.00 (0.86, 1.16)	0.93 (0.80, 1.08)	0.22

Dietary DHA and EPA adjusted for energy using the residual method. CI: Confidence interval. HR: Hazard ratio. Model 1: adjusted for age, sex, and race; Model 2: adjusted for center, age, race, sex, energy intake, BMI, education, exercise levels, smoking status and amount, alcohol intake, HDL-C, LDL-C, use of cholesterol lowering medications, systolic blood pressure, use of antihypertensive medications, diabetes, coronary heart disease, and ECG-defined left ventricular hypertrophy.

### Risk of AF by Phospholipid Levels of DHA and EPA

Among the 3,757 eligible ARIC participants from the Minneapolis center with measurements of phospholipid fatty acids, 401 AF events occurred during an average follow-up time of 17.9 years (67,081 person-years).

In multivariable analyses, the test for linear trend across quartiles of phospholipid DHA+EPA for incident AF was not statistically significant (table 4). When analyzed separately, DHA showed a marginal U-shaped association (p for quadratic term = 0.10) with highest risk in the first quartile and in the last quartile, lowest in the middle quartiles, and with point estimates below 1.00 for intakes greater than the first quartile (table 4). In contrast, EPA showed no association with the incidence of AF (Q4 vs. Q1, HR = 1.12, 95% CI: 0.85–1.49).

**Table 4:** Hazard ratios (95% confidence interval) of atrial fibrillation by quartiles of Phospholipid DHA and EPA, ARIC Minnesota field center, 1987–2008.

Phospholipid DHA+EPA (Quartiles)	Q1	Q2	Q3	Q4	P for trend
AF cases	112	95	93	101	
Person-years	16,114	16,994	16,829	17,144	
Model 1 [HR (95% CI)]	1 (ref.)	0.77 (0.58, 1.01)	0.80 (0.61, 1.05)	0.79 (0.60, 1.03)	0.18
Model 2 [HR (95% CI)]	1 (ref.)	0.80 (0.6, 1.06)	0.81 (0.61, 1.08)	0.87 (0.66, 1.15)	0.54

Phospholipid DHA From Plasma (Quartiles)	Q1	Q2	Q3	Q4	
AF cases	117	86	99	99	
Person-years	16,118	16,961	16,849	17,153	
Model 1 [HR (95% CI)]	1 (ref.)	0.68 (0.52, 0.90)	0.78 (0.60, 1.02)	0.74 (0.57, 0.97)	0.10
Model 2 [HR (95% CI)]	1 (ref.)	0.71 (0.54, 0.95)	0.82 (0.62, 1.08)	0.84 (0.63, 1.11)	0.47

Phospholipid EPA (Quartiles)	Q1	Q2	Q3	Q4	
AF cases	99	86	106	110	
Person-years	17,325	15,505	17,217	17,034	
Model 1 [HR (95% CI)]	1 (ref.)	1.00 (0.75, 1.34)	1.11 (0.84, 1.46)	1.11 (0.85, 1.46)	0.38
Model 2 [HR (95% CI)]	1 (ref.)	0.98 (0.73, 1.31)	1.04 (0.78, 1.37)	1.12 (0.85, 1.49)	0.33

CI: Confidence interval. HR: Hazard ratio. Model 1: adjusted for age and sex; Model 2: adjusted for age, sex, BMI, education, exercise levels, smoking status and amount, alcohol intake, HDL-C, LDL-C, use of cholesterol lowering medications, systolic blood pressure, use of antihypertensive medications, diabetes, coronary heart disease, and ECG-defined left ventricular hypertrophy.

### Risk of AF by Combined Dietary and Biomarker Categories

Among the 3,743 ARIC participants from the Minneapolis center with measurements of phospholipid fatty acids and dietary fatty acid intake, 400 AF events occurred during an average follow-up of 17.9 years (66,834 person-years).

Participants with both dietary intake and phospholipid levels of DHA+EPA in the highest tertiles did not have significantly different risk of AF compared with those in the lowest tertiles (table 5). Howe's method with three categories yielded similar results as well (table 5). Finally, Howe's method with n categories (n = 3,743) did not provide any evidence of an association between n-3 PUFA intake and AF risk. Comparing the estimated hazard for the subject with the lowest intake of DHA+EPA (rank = 1) with the highest (rank = 3,745) yielded a hazard ratio of 0.92 (95% CI: 0.74–1.14). Results were similar when exposure was restricted to intake of oily fish and canned tune, and biomarker data on DHA (data not shown).

**Table 5:** Hazard ratio (95% confidence interval) of atrial fibrillation by combined dietary and biomarker DHA and EPA, ARIC Minnesota field center, 1987–2005.

Tertiles	Both Lowest Tertile	Other	Both Highest Tertile			P for trend
AF cases	78	255	67			
Person-years	11,708	43,270	11,856			
HR (95% CI)*	1.0 (ref.)	0.95 (0.72–1.24)	0.98 (0.69–1.39)			0.91

Howe's Method 3 –Categories	Rank = 2	Rank = 3	Rank = 4	Rank = 5	Rank = 6	P for trend
AF cases	78	76	102	77	67	
Person-years	11,708	13,327	15,942	14,000	11,856	
HR (95% CI)*	1.0 (ref.)	0.93 (0.68, 1.29)	1.00 (0.73, 1.36)	0.90 (0.64, 1.26)	0.98 (0.69, 1.39)	0.83

* Adjusted for age, sex, BMI, education, energy intake, exercise levels, smoking status and amount, alcohol intake, total cholesterol, use of cholesterol lowering medications, systolic blood pressure, use of antihypertensive medications, diabetes, coronary heart disease, and ECG-defined left ventricular hypertrophy.

CI: Confidence interval. HR: hazard ratio.

## Discussion

In this population-based study of middle-aged adults, we did not observe strong associations of fish intake or dietary EPA and DHA with risk of AF. However, our results suggest that the association between fish-derived phospholipid n-3 PUFAs may differ for individual fatty acids.

Overall, our results are consistent with those from the Women's Health Initiative [8], the Rotterdam Study [9], the Framingham Heart Study [13], and the Danish Diet, Cancer, and Health Study [10]: four large prospective cohorts that also failed to observe an association between fish intake and AF risk. Only one previous analysis in the Cardiovascular Health Study, involving a cohort of Americans aged 65 years and older, found that higher consumption of tuna or other non-fried fish was associated with a 30% lower risk of AF (5+ servings/week vs. <1 serving/month, RR = 0.70, 95% CI: 0.53–0.93) [12]. Notably, the Cardiovascular Health Study had a large proportion of subjects with high fish consumption (19% ate 5+ servings/week of tuna or other fish vs. 8.3% in ARIC), suggesting that a higher intake than that observed in the present study might be necessary to illicit any effect on AF risk as has been observed for other cardiovascular outcomes [12], [27]. Our finding that higher intake of oily fish and canned tuna in the ARIC cohort was associated with a lower, though non-significant, risk of AF is consistent with the results from the Cardiovascular Health Study.

Our data suggested that phospholipid EPA and DHA may be differentially associated with AF risk, with no association between EPA levels and AF risk and a U- or L-shaped association between DHA and AF risk. Two previous studies have explored the associations between blood levels of DHA and EPA—as biomarkers of fish intake—and AF risk. A Finnish prospective study found an inverse association between blood serum levels of total fish-derived n-3 PUFAs (RR = 0.50, 95% CI: 0.31–0.80, comparing extreme quartiles) and DHA (RR = 0.51, 95% CI: 0.32–0.82, comparing extreme quartiles) with incident AF, but no association was seen for other n-3 PUFAs (EPA, docosapentaenoic acid) [14]. Very similar results were found in the Cardiovascular Health Study [16]. ARIC results are somewhat consistent with these two studies, though fish intake in both studies was higher than that observed in the ARIC cohort. In ARIC subjects, the highest DHA+EPA quartile contained values >3.83% whereas Finland's Kuopio Ischemic Heart Disease Risk Factor Study had values <3.61% in the lowest quartile and >5.33% in the highest [14], and average total n-3 PUFA concentration in the Cardiovascular Health Study was 4.5%. In addition, a recent small case-control study in Italy found that levels of n-3 PUFA in erythrocyte membranes were higher in cases of idiopathic AF than in controls (31.4% vs. 23.5%, p<0.001) [15], but this later study did not adjust for potential confounders and, therefore, its interpretation is problematic.

Measurement error may be partly responsible for the inconsistent results across studies. Phospholipid measurement of n-3 fatty acids, less subject to self-report measurement error, could better elucidate the relationship between n-3 fatty acids and AF. However, our results combining phospholipid and dietary data were similarly null.

Other reasons for why certain subgroups show an inverse association between n-3 PUFAs and AF include dose and relevant exposure period. Some trials [28], [29], [30] (but not all [31], [32]) have shown that fish oil supplementation pre- and post-coronary artery bypass graft (CABG) or open heart surgery reduces the risk of AF—especially in the immediate aftermath [29]. These studies used n-3 PUFA doses that exceed typical dietary intake, and were done in subjects at high risk for AF. Similarly, trials investigating n-3 PUFA supplementation once AF has been established have also shown mixed results [33], [34]. This may suggest that the administration of high-dose n-3 PUFA supplementation (as opposed to usual intake) in high-risk subjects might have a preventive effect, though further research is required.

In spite of the lack of strong and consistent epidemiologic evidence for an inverse association of n-3 PUFAs with AF, several mechanisms suggest that such an effect might exist. First, fish-derived n-3 PUFAs may prevent AF by preventing the structural heart damage that is a precursor to AF. Previous research has shown that fish-derived n-3 PUFAs protect against CHD [1], [2], a risk factor for AF [35]. Second, n-3 PUFAs may inhibit the inflammatory triggers that occasionally initiate the ectopic activity in AF [36], [37]. Finally, even once that electrical activity has been stimulated, fish-derived n-3 PUFAs may inhibit the fast, voltage-dependent sodium current and the L-type calcium currents [38], [39] that would allow the arrhythmia to be sustained.

Our study is not without limitations. Data were not available on fish preparation technique. Analysis in the Cardiovascular Health Study have shown that fish preparation method differentially effects the association between fish-derived n-3 PUFAs and CHD, with only intake of tuna and other baked or broiled fish associated with cardiovascular benefits, with no or deleterious associations for fried fish or fish sandwiches [2], [40]. Lack of information on fish preparation method in ARIC could be responsible for our failure to show an association between fish intake and AF risk. The relatively low correlation between dietary and plasma EPA and DHA in the ARIC sample further suggests that measurement error is an important limitation.[23] Additionally, the range of fish intake in our study sample was low. Such a limited range may have inhibited our ability to find an association. Also, diet was measured at baseline and at a follow-up visit, but changes in fish consumption after visit 3 could not be assessed, potentially creating additional nondifferential measurement error of the exposure. Finally, AF ascertainment was mostly based on hospital discharge codes, which limited the ability to identify paroxysmal AF and AF managed in outpatient settings. Our previous validation study, however, suggests that hospital discharges are an adequate method for AF ascertainment in epidemiologic studies [20].

In summary, our results suggest that, in a sample with low-moderate average fish intake, usual fish and n-3 PUFAs intake is not strongly associated with AF risk. We have observed, consistent with other studies [14], that phospholipid DHA and EPA might have dissimilar associations with the risk of AF.
